# Microbial Contamination, Degradation Characteristics of Dominant Bacteria on the Hull of the Nanhai No. 1 Shipwreck

**DOI:** 10.3390/ijms27125631

**Published:** 2026-06-22

**Authors:** Yu Wang, Yeqing Han, Cen Wang, Zeao Wang, Zhiqian Guan, Naisheng Li, Jiao Pan

**Affiliations:** 1 Key Laboratory of Archaeomaterials and Conservation, Ministry of Education, University of Science and Technology Beijing, Beijing 100083, China; d202310766@xs.ustb.edu.cn (Y.W.);; 2 Institute for Cultural Heritage and History of Science & Technology, University of Science and Technology Beijing, Beijing 100083, China; 3 College of Life Sciences, Nankai University, Tianjin 300071, China; 4 National Centre for Archaeology, Beijing 100013, China

**Keywords:** Nanhai No. 1 shipwreck, wood degradation, microbial community analysis, *Brevibacterium* sp., enzyme activity

## Abstract

To clarify the microbial contamination and wood degradation risk of the Nanhai No. 1 shipwreck hull and verify on-site antibacterial agent effectiveness, microbial samples were collected and analyzed via SEM, metagenomic sequencing, bacterial isolation, enzyme activity detection, and antibacterial experiments. The results showed that Actinomycetota was the dominant phylum, and *Brachybacterium*, *Microbacterium*, and *Brevibacterium* were the dominant genera. Seven bacterial strains were isolated and purified, among which *Brevibacterium* sp. (NH.SH-B6) had the strongest wood degradation ability, possessing cellulase, LiP, MnP, and Lac activities. When cultured with hull wood as the sole carbon source, LiP was the dominant degrading enzyme of NH.SH-B6, and its maximum enzyme activity was achieved under the optimal conditions of pH = 7, 10% NaCl, 1000 mg/L FeSO_4_, and no PEG400 added. 50 mg/mL cinnamaldehyde and 0.5% isothiazolinone K100 had good inhibitory effects on the isolated bacteria, and bacterial proliferation was due to incomplete antibacterial agent spraying. This study clarifies the microbial degradation risk of the Nanhai No. 1 shipwreck hull and provides a scientific basis for optimizing the on-site protection strategy of the shipwreck.

## 1. Introduction

As pivotal carriers of ancient human civilization, wooden cultural relics exist in diverse forms such as ships, coffins, and architectural components, serving as invaluable physical evidence for investigating ancient history, art, science and technology, and economic systems [1,2,3]. Among them, marine-excavated wooden artifacts, as a typical category of waterlogged wooden cultural relics, have undergone prolonged immersion in marine environments, developing distinct characteristics: extremely high moisture content, severe wood degradation, elevated salt levels (including both soluble salts and insoluble iron-sulfur compounds), and high susceptibility to microbial deterioration, which render their long-term preservation and protection exceptionally challenging [4,5,6,7,8]. Beyond encapsulating critical insights into ancient navigation technology and transregional trade, these relics serve as direct corroboration of cultural exchanges along the Maritime Silk Road, holding irreplaceable historical and scientific value [9,10].

The Nanhai No. 1 ancient shipwreck, a wooden merchant vessel that sank during a cargo voyage along the Maritime Silk Road in the Southern Song Dynasty (1127–1279 AD), is a representative of such marine-excavated wooden relics [11,12]. Salvaged intact in 2007 and transferred to the Guangdong Maritime Silk Road Museum for long-term preservation, it is the largest, best-preserved, and most relic-laden ocean-going merchant ship unearthed in China’s archaeological record, with a remaining length of 22.95 m, a maximum preserved width of 9.85 m, a maximum internal cabin depth of 2.7 m, and an overall height exceeding 3 m. After nearly 800 years of marine burial, the hull wood has suffered moderate-to-severe degradation. Additionally, the wood contains substantial concentrations of salts [13].

Since the formal launch of archaeological excavations in 2013, protective measures have been implemented to preserve the hull, including automatic sprinkler systems supplemented with non-woven fabric coverings to maintain moisture levels and prevent dehydration-induced deformation, as well as biocides added to the sprinkler solution to mitigate microbial deterioration. However, the hull’s complex structure creates blind spots in the sprinkler system, and the persistent high-humidity environment fosters microbial proliferation, leading to visible microbial plaques on the hull surface that pose a severe threat to the structural stability of the hull wood [14]. Microbial deterioration constitutes one of the core challenges in protecting marine-excavated wooden cultural relics. Driven by the metabolic activities of microorganisms (including bacteria, fungi, and algae), biological deterioration induces alterations in the physical and chemical properties of relic materials, causes structural damage, and in severe cases, results in irreversible degradation [15,16,17,18]. Research on microbial deterioration of the Nanhai No. 1 has focused predominantly on fungal communities; fungi such as *Fusarium solani* have been identified as dominant deteriorative fungi, efficiently degrading wood cellulose and lignin through the secretion of degrading enzymes, including lignin peroxidase and laccase [19,20,21,22]. In contrast, research on the role of bacteria in hull degradation remains insufficient, with the compositional characteristics, degradation potential, and adaptive mechanisms of bacterial communities yet to be systematically elucidated.

Notably, bacteria play a non-negligible role in the biological degradation of wooden cultural relics. In aquatic environments, Erosion Bacteria and Tunneling Bacteria are the primary microbial groups responsible for degrading waterlogged wood: the former efficiently degrades cellulose under hypoxic conditions, forming erosion furrows on wood cell walls, while the latter decomposes cell wall polysaccharides and lignin, leading to wood softening and discoloration [15,16,23,24]. Furthermore, a wide range of bacteria can produce degrading enzyme systems (cellulase, lignin peroxidase, and manganese peroxidase) that mediate the decomposition of key wood components [25,26,27]. Worldwide studies on wooden relic biodeterioration show that halotolerant microbes dominate wood decay in marine environments. Tropical marine research has also clarified microbial stress adaptation and relevant conservation approaches [28,29,30,31]. These works establish a sound theoretical foundation for investigations into waterlogged wood deterioration. For instance, in the conservation of renowned marine-excavated relics such as the British Mary Rose and Swedish Vasa shipwrecks, abundant bacterial communities with degradation potential (including *Bacillus*, *Micrococcus* and *Acinetobacter*) have been detected, whose metabolic activities have accelerated hull wood corrosion [32,33].

The unique microenvironment of the Nanhai No. 1 hull, characterized by low pH, high salinity, and elevated iron-sulfur compound levels, offers a distinct ecological niche for bacterial colonization and proliferation. The crystallization-dissolution cycles of soluble salts accelerate cellulose degradation and fragmentation, while the oxidation of iron-sulfur compounds to sulfuric acid induces wood acidification, likely selecting for bacterial taxa with specialized adaptive traits [34]. Additionally, atmospheric oxygen exposure during excavation can alter the microbial community structure, favoring aerobic or facultatively anaerobic bacteria [35,36]. Against this backdrop, the current on-site protection measures for the Nanhai No. 1 face limitations due to sprinkler blind spots, resulting in persistent microbial contamination in some areas. Crucially, gaps remain in understanding the hull’s bacterial community composition, dominant degrading bacterial strains, their degradation mechanisms, and the key factors affecting their degrading enzyme activity, limiting the formulation of targeted and efficient protection strategies.

In view of these problems, this study conducted a systematic investigation of microbial contamination on the Nanhai No. 1 shipwreck. Using scanning electron microscopy, metagenomic sequencing, bacterial isolation and purification, molecular identification, enzyme activity detection, and laboratory antibacterial experiments, we aimed to clarify the hull’s microbial community structure, identify dominant degrading bacterial strains, explore their degradation characteristics and the influence of culture conditions on their degrading enzyme activity, and verify the effectiveness of existing on-site antibacterial agents. The research results are expected to fill gaps in current research on microbial contamination of the Nanhai No. 1, provide a scientific basis for optimizing on-site protection measures, and contribute to the long-term effective preservation of this precious cultural relic.

## 2. Results

### 2.1. SEM Observation and Microbial Diversity Analysis on the Hull of the Nanhai No. 1 Shipwreck

**Figure 1:**
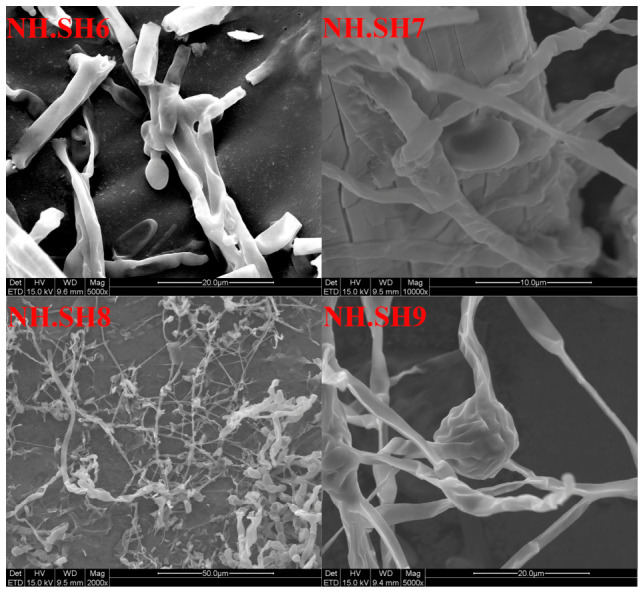
SEM observation results of the hull surface of the Nanhai No. 1 shipwreck.

**Figure 2:**
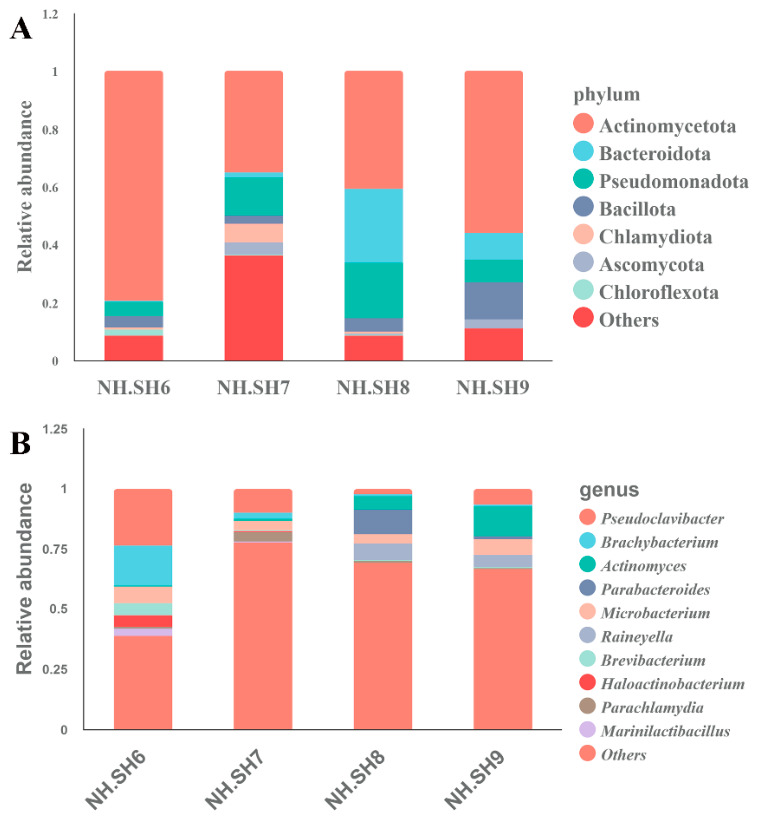
Richness of microbial species analysis results of the hull surface of the Nanhai No. 1 shipwreck. (**A**) Relative abundance of microorganisms at the phylum level; (**B**) Relative abundance of microorganisms at the genus level.

Scanning electron microscopy (SEM) was performed on microbially contaminated samples obtained from four representative sites (NH.SH6–NH.SH9) on the wooden hull surface of the Nanhai No. 1 shipwreck. The SEM micrographs exhibited obvious microbial colonization and attachment characteristics on the hull surface. Typical microbial morphologies, including fungal hyphae structures, were clearly observed in the samples. These intuitive morphological results directly demonstrated that microorganisms successfully colonized the shipwreck hull surface, which further aggravates the biodeterioration of ancient wood relics (Figure 1). Metagenomic sequencing was performed on these four samples, and microbial community composition analysis was carried out based on the sequencing data. At the phylum level, Actinomycetota exhibited the highest average relative abundance, accounting for 52.78%. In addition, Bacteroidota, Pseudomonadota, Bacillota, and Ascomycota also showed relatively high average relative abundances, with their relative contents reaching 9.21%, 11.22%, 6.10%, and 1.92%, respectively (Figure 2A). At the genus level, the top ten microorganisms with the highest abundance were all bacteria (Figure 2B). Among these genera, those with an average relative abundance greater than 1% included *Pseudobacterium* (10.65%), *Brachybacterium* (5.29%), *Microbacterium* (5.13%), *Actinomyces* (4.72%), *Raineyella* (2.92%), *Paraacteroides* (2.90%), *Brevibacterium* (1.80%), *Haloactinnobacterium* (1.38%), and *Parachlamydia* (1.33%).

### 2.2. Microbial Function Annotation Analysis

**Figure 3:**
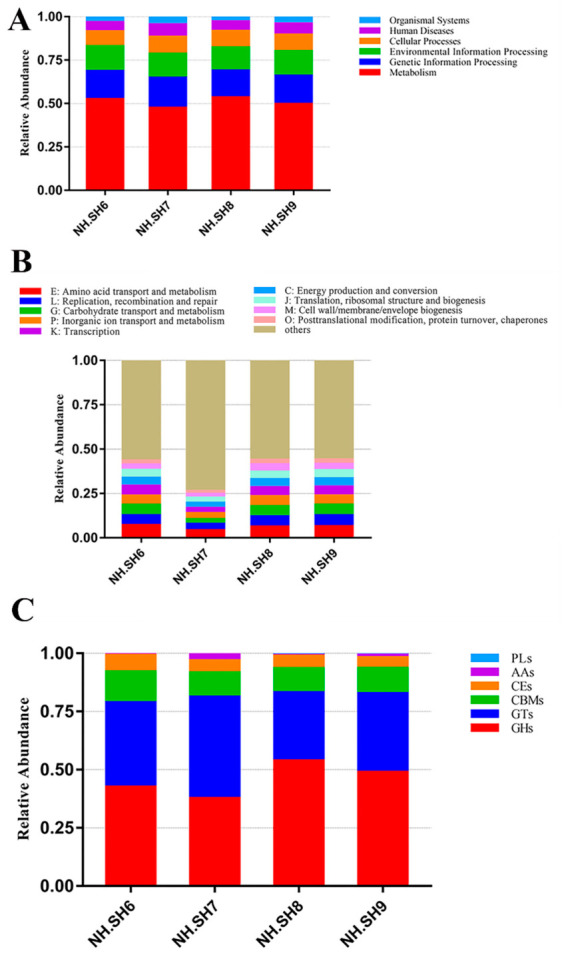
Microbial function annotation analysis results. (**A**) Relative abundance of KEGG database at level 1 in metagenome sequencing; (**B**) Relative abundance of eggNOG database at level 1 in metagenome sequencing; (**C**) Relative abundance of CAZy database in metagenome sequencing.

The sequencing results were analyzed using various functional databases to explore the degradation mechanism of microorganisms on the ship hull wood. The level 1 annotation results of the KEGG database showed that the gene abundance in the metabolic pathway was the highest (Figure 3A). Annotations related to wood degradation were analyzed at different levels: at the KO level, β-Glucosidase (K05349) was identified with an average relative abundance of 0.04%; at the module level, the average relative abundance of pectin degradation (M00081) was 0.0015%. The level 1 annotation results of the eggNOG database indicated that the top three categories in terms of relative abundance were amino acid transport and metabolism, reproduction, recombination and repair, and carbohydrate transport and metabolism (Figure 3B). For the CAZy database, the level 1 annotation results showed that Glycoside Hydrolases (GHs) had the highest abundance (Figure 3C); notably, GHs contain a variety of enzymes related to wood degradation, such as β-Glucosidases and exo-1,3-1,4-Glucanases.

### 2.3. Determination of Biodeterioration of Hull Wood by Major Bacteria

**Figure 4:**
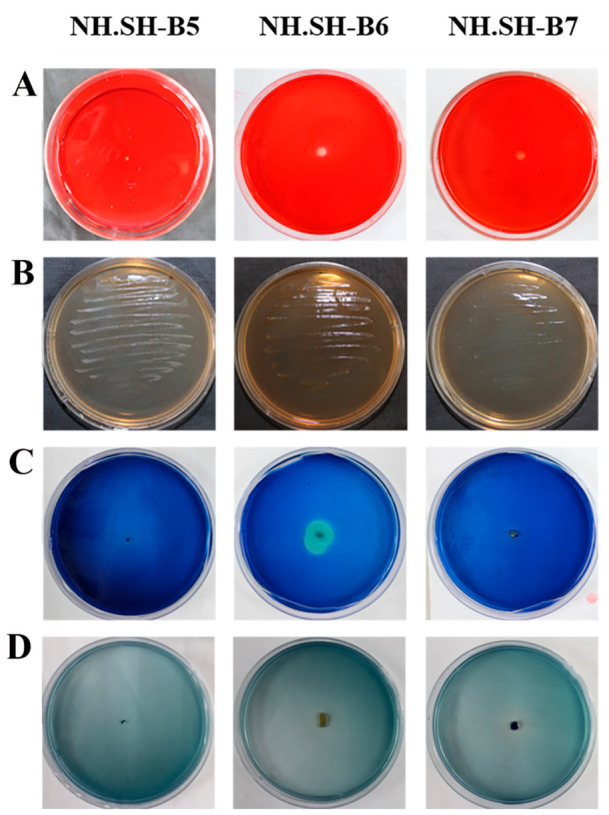
Preliminary detection results of cellulose and lignin degradation capabilities of major bacterial strains. (**A**) Cellulose degradation capability on CMC-Na agar medium; (**B**) Lignin degradation capability on sodium lignosulfonate medium; (**C**) LiP and MnP activities on aniline blue medium; (**D**) Lac activity on Remazol brilliant blue medium.

Seven bacterial strains were isolated and purified from the hull of the Nanhai No. 1 shipwreck (Table 1). Among these strains, *Brachybacterium paragonglomeratum* (NH.SH-B5), *Brevibacterium* sp. (NH.SH-B6), and *Brevundimonas* sp. (NH.SH-B7) exhibited relatively high relative abundances in metagenomic sequencing, ranking 2nd (5.29%), 8th (1.8%), and 26th (0.38%), respectively. Preliminary tests were performed to evaluate the cellulose and lignin degradation capabilities of these three bacterial strains. The results showed that NH.SH-B6 formed the most distinct transparent zone on CMC-Na agar medium, indicating a strong ability to degrade cellulose (Figure 4A). Both NH.SH-B5 and NH.SH-B6 were able to grow on sodium lignosulfonate medium, demonstrating their capacity to grow and reproduce using lignin as the sole carbon source (Figure 4B). On aniline blue medium, NH.SH-B6 produced the most obvious transparent zone, suggesting its ability to secrete LiP and MnP (Figure 4C). In addition, NH.SH-B6 and NH.SH-B7 formed relatively distinct transparent zones on Remazol brilliant blue medium, indicating their ability to produce Lac (Figure 4D). Collectively, these results indicated that among the cultivable bacterial strains, *Brevibacterium* sp. (NH.SH-B6) possessed cellulase, LiP, MnP, and Lac activities, making it the most threatening bacterium for the biodeterioration of ship hull wood.

### 2.4. Effects of Different Culture Conditions on Enzyme Activity of NH.SH-B6

**Figure 5:**
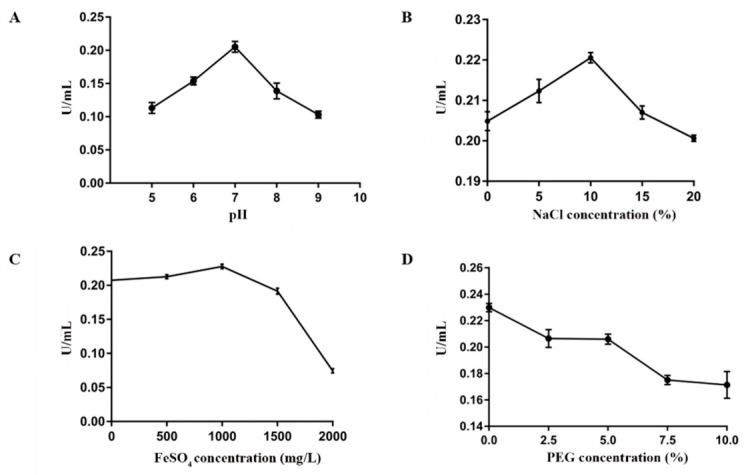
Variation trend of LiP enzyme activity of NH.SH-B6 under different culture conditions. (**A**) Effect of pH on LiP enzyme activity; (**B**) Effect of NaCl concentration on LiP enzyme activity; (**C**) Effect of FeSO_4_ concentration on LiP enzyme activity; (**D**) Effect of PEG400 concentration on LiP enzyme activity.

The activities of cellulose and lignin-degrading enzymes of the bacterium NH.SH-B6 were determined. The results showed that when cultured with the ancient sunken wood of the Nanhai No. 1 shipwreck as the sole carbon source, the dominant degrading enzyme of NH.SH-B6 was LiP, with a maximum enzyme activity of 0.1953 U/mL and a peak production time of 54 h (Table 2). To investigate the effects of various factors on the LiP activity of NH.SH-B6, different concentration gradients of pH value, NaCl, FeSO_4_, and PEG400 were established. The results indicated that the LiP activity of NH.SH-B6 was the highest (0.2054 U/mL) when the pH value was 7 (Figure 5A). Regarding the effect of NaCl concentration on LiP activity, under the condition of pH = 7, the highest LiP activity of NH.SH-B6 (0.2206 U/mL) was observed when the NaCl concentration was 10% (Figure 5B). For the effect of FeSO_4_ concentration, under the optimal conditions of pH = 7 and 10% NaCl, the LiP activity of NH.SH-B6 reached the maximum (0.2278 U/mL) when the FeSO_4_ concentration was 1000 mg/L (Figure 5C). As for the effect of PEG400 concentration, the highest LiP activity of NH.SH-B6 (0.2299 IU/mL) was obtained in the absence of PEG400 (Figure 5D). Collectively, when cultured with hull wood as the sole carbon source, the maximum LiP activity of NH.SH-B6 was achieved under the following optimal conditions: pH = 7, NaCl concentration of 10%, FeSO_4_ concentration of 1000 mg/L, and no PEG400 added.

### 2.5. Results of Laboratory Antibacterial Experiments

**Figure 6:**
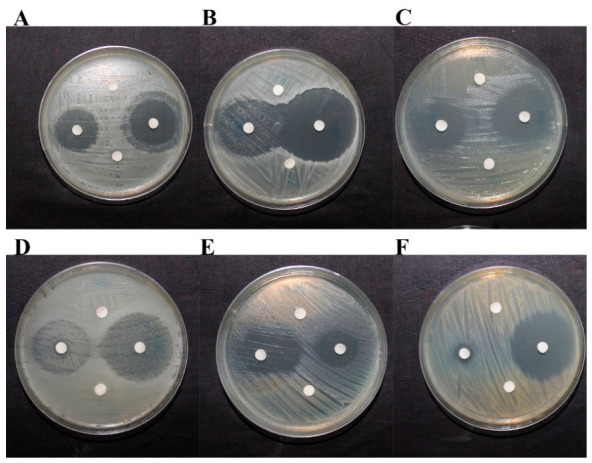
Results of antibacterial experiments. The filter paper disc on the upper side was the negative control (ddH_2_O), the one on the lower side was the negative control (DMSO), the one on the left side was 50 mg/mL cinnamaldehyde, and the one on the right side was 0.5% K100. A larger inhibition zone diameter indicates stronger antibacterial activity. (**A**) NH.SH-B1; (**B**) NH.SH-B2; (**C**) NH.SH-B3; (**D**) NH.SH-B4; (**E**) NH.SH-B5; (**F**) NH.SH-B6.

Under laboratory conditions, the inhibitory effects of 50 mg/mL cinnamaldehyde and 0.5% isothiazolinone K100 on the isolated bacterial strains were tested. The results showed that both antibacterial agents exhibited good inhibitory effects on the bacteria (Figure 6), indicating that the two antibacterial agents currently used in the on-site protection of the Nanhai No. 1 shipwreck are effective. The main reason for bacterial proliferation is that the antibacterial agents cannot be sprayed onto the sampling sites.

## 3. Discussion

As a precious large-scale wooden cultural relic, the preservation state of the hull of the Nanhai No. 1 shipwreck is directly related to the inheritance of the cultural relic’s historical value, and biodeterioration caused by microbial contamination is the core problem threatening the stability of the hull’s wooden structure. Based on the investigation of microbial contamination on the hull of the Nanhai No. 1 shipwreck and a series of subsequent experiments, this study systematically explored the characteristics of the hull’s microbial community, the degradation properties of dominant pathogenic strains, and the effectiveness of existing antibacterial measures. It provides a targeted scientific basis for shipwreck protection and fills the gap in the current research on microbial contamination of this shipwreck regarding the degradation mechanism of dominant strains and the regulation of culture conditions.

Metagenomic sequencing results showed that Actinomycetota was the dominant phylum in the microbial community on the hull surface, with an average relative abundance of as high as 52.78%, followed by Bacteroidota, Pseudomonadota, etc. This is basically consistent with the characteristics of the microbial community composition of waterlogged wooden cultural relics in aquatic environments, suggesting that the formation of this community structure is closely related to the hull’s long-term preservation in a high-humidity and specific-salinity environment [6,37,38]. Among the 7 bacterial strains isolated from the hull, *Brevibacterium* sp. (NH.SH-B6) ranked 8th in relative abundance (1.8%) in metagenomic sequencing. Preliminary screening of lignin and cellulose degradation capabilities confirmed that this strain can not only grow on the medium with lignin as the sole carbon source but also produce cellulase, lignin peroxidase, manganese peroxidase, and laccase. It is the most threatening pathogenic strain to hull wood degradation among all cultivable strains [39,40,41]. This result clarifies the core dominant pathogenic bacteria responsible for the biodeterioration of the Nanhai No. 1 shipwreck hull and provides a key target for subsequent targeted prevention and control.

Comparing our results with previous studies on marine wooden relics worldwide, obvious similarities and differences can be observed. Most related investigations have identified *Bacillus* and *Acinetobacter* as the predominant wood-degrading bacteria on ancient ship hulls, and reported that Pseudomonadota frequently dominates microbial communities in marine environments [31,32,33]. Distinct from those findings, the major degrading strain isolated in this study belongs to Actinomycetota, a taxon commonly found in high-salt marine habitats. Furthermore, most existing studies mainly focus on microbial community composition under natural marine conditions, whereas our work further characterizes the enzyme activity of functional strains under simulated in situ environments. Previous research on the Nanhai No. 1 shipwreck primarily concentrated on fungal groups [19,20,21,22]. By focusing on bacteria, the present study complements the existing research system and overcomes the limitations of previous research perspectives.

Culture experiments using the ancient sunken wood of the Nanhai No. 1 shipwreck as the sole carbon source showed that LiP was the dominant degrading enzyme of NH.SH-B6, which is consistent with the research conclusion that lignin peroxidase serves as the core degrading enzyme in the degradation process of wooden cultural relics. This indicates that the strain mainly dominates the degradation of hull lignin by secreting LiP, thereby damaging the integrity of the wooden structure. Experiments on the effects of different culture conditions on LiP activity showed that the LiP activity reached the highest level (0.2299 IU/mL) when pH = 7, NaCl concentration was 10%, FeSO_4_ concentration was 1000 mg/L, and no PEG400 was added. This result is highly consistent with the current preservation environment characteristics of the hull: the high-salt environment (close to 10% NaCl concentration) and sulfur-rich iron compound matrix conditions of the hull just provide a suitable environment for the secretion of LiP by NH.SH-B6, further explaining the reason for the continuous occurrence of microbial degradation on the hull. However, the addition of PEG400 can inhibit LiP activity, suggesting that if PEG400 is used for wood reinforcement during shipwreck protection, its impact on microbial degrading enzyme activity should be taken into account to avoid increasing the risk of hull degradation.

The results of laboratory antibacterial experiments confirmed that 50 mg/mL cinnamaldehyde and 0.5% isothiazolinone K100 used in on-site protection had good inhibitory effects on all isolated pathogenic bacteria, indicating that the currently selected antibacterial agents themselves are effective. The core reason for microbial contamination in some areas of the hull is the blind spots in the spraying system caused by the complex structure of the hull, which prevents the antibacterial agents from fully covering the hull, rather than the ineffectiveness of the antibacterial agents themselves. This finding corrects the potential cognitive bias that antibacterial agents are ineffective and provides an important direction for optimizing the shipwreck protection strategy: there is no need to replace the antibacterial agents; the focus should be on improving the spraying method, expanding the spraying coverage, reducing spraying blind spots, while strengthening microbial monitoring of blind areas and taking timely supplementary antibacterial measures.

Comprehensive analysis of all experimental results in this study shows that the microbial degradation of the Nanhai No. 1 shipwreck hull is mainly dominated by pathogenic bacteria represented by NH.SH-B6, and its degradation ability is significantly affected by the hull’s own preservation environment (pH, salinity, and iron compound content). The core problem of current antibacterial measures lies in insufficient spraying coverage. Based on this, the subsequent shipwreck protection work should focus on three aspects: first, regularly monitor the changes in the hull’s microbial community and the quantity dynamics of NH.SH-B6 to timely grasp the degradation risk; second, optimize the spraying system to reduce blind spots and ensure uniform coverage of the hull surface by antibacterial agents; third, combine the optimal LiP activity conditions of NH.SH-B6 to targetedly regulate the hull’s preservation environment, inhibit the activity of dominant degrading enzymes, and reduce the biodeterioration rate. This study clarifies the key mechanisms and influencing factors of microbial degradation of the Nanhai No. 1 shipwreck hull, and provides a direct experimental basis and a feasible technical reference for the long-term effective protection of this shipwreck.

## 4. Materials and Methods

### 4.1. Investigation and Sample Collection of Microbial Contamination on the Nanhai No. 1 Shipwreck

**Figure 7:**
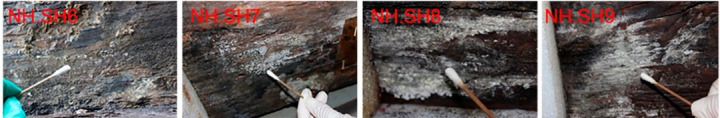
Sampling of microbial contamination.

An investigation into microbial contamination of the Nanhai No. 1 shipwreck was carried out. The results showed that white microbial contamination plaques were present in some areas on the hull surface. Four sampling sites (NH.SH6-NH.SH9) were set on the hull surface for sample collection in the same month (Figure 7). All sampling sites were located on the deck of the Nanhai No. 1 shipwreck, which was arranged adjacent to the hull. The spraying system could only cover partial areas of the hull, and the sampling sites were not within the spraying range. The preservation environment had an average annual temperature of 25.6 °C and an average annual relative humidity of 84.1%.

Microbial sampling was performed following three specific procedures: (1) Microbes on the surface of microbial contaminated surfaces were adhered using double-sided carbon conductive tape, which was then placed in sterilized EP tubes for subsequent scanning electron microscopy (SEM) observation; (2) Microbial samples were inoculated onto LB solid medium for the isolation, purification, and molecular identification of major bacteria; (3) The surface of microbial contaminated surfaces was gently scraped with a sterilized scalpel, and the scraped samples were collected into sterilized EP tubes for total DNA extraction and metagenomic sequencing. All the collected samples were stored in an ice box and transported back to the laboratory under low-temperature conditions for subsequent experimental operations.

### 4.2. SEM Observation

Microbial samples from the surface of the Nanhai No. 1 shipwreck were adhered using carbon conductive tape and then dried in a desiccator. After drying, the samples were attached to the SEM sample stage. Gold sputtering was performed at a current of 24 mA for 300 s, followed by SEM (CX-200K, Tokyo, Japan) observation and image recording. The measurement conditions were set as follows: EHT: 15.0 kV, WD: 9.4–9.6 mm, Mag: 2KX-10KX.

### 4.3. Metagenomic Sequencing Analysis

Total DNA was extracted using the DNeasy PowerSoil Kit (QIAGEN, Hilden, Germany, Cat. No. 47014) and then sent to NovoMagic Technology Co., Ltd. (Beijing, China) for sequencing on the Illumina HiSeq sequencing platform. Readfq (https://github.com/cjfields/readfq, accessed on 10 December 2020) was used for preprocessing raw data from the Illumina sequencing platform to obtain clean data for subsequent analysis. The specific steps are as follows: (a) Remove reads with low-quality bases (default quality threshold is ≤38) over a length of 40 bp; (b) Remove reads containing ambiguous N bases exceeding 10 bp; (c) Remove reads with adapter overlapping regions longer than 15 bp. Subsequently, MEGAHIT software (Version 1.0.4) was adopted to assemble the clean data with the parameters --presets meta-large (--end-to-end, --sensitive, -I 200, -X 400). The resulting scaffolds were split at N junctions to generate N-free scaftigs. With default parameters, MetaGeneMark (http://topaz.gatech.edu/GeneMark, accessed on 10 December 2020) was applied to predict open reading frames (ORFs) for scaftigs longer than or equal to 500 bp. Predicted sequences shorter than 100 nt were further filtered out. CD-HIT software (Version 4.5.8) was then used to remove redundant ORFs with the parameters -c 0.95, -G 0, -aS 0.9, -g 1, -d 0, generating a non-redundant initial gene catalogue. Bowtie2 (Version 2.2.4) was used to map clean reads of each sample against the initial gene catalogue with the parameters --end-to-end, --sensitive, -I 200, -X 400, and the number of reads mapped to each gene was counted. Genes with no more than 2 mapped reads per sample were discarded to obtain the final gene catalogue (Unigenes). Gene abundance was calculated based on the number of mapped reads and gene length for data normalization. DIAMOND software (Version 0.9.9.110) was used to align Unigene sequences with those of bacteria, fungi, archaea, and viruses extracted from the NCBI NR database (Version 2020-12-10). Additionally, DIAMOND software (Version 0.9.9) was employed to align Unigenes with sequences in functional databases, including the KEGG database (Version 2020-12-10), eggnog database (Version 4.5), and CAZy database (Version 202012). From the alignment results of each sequence, the Best Blast Hit results were selected for subsequent analysis. The raw sequencing data are available in the NCBI Sequence Read Archive (SRA) with the study accession number PRJNA1015580.

### 4.4. Isolation, Purification and Molecular Identification of Bacteria

For bacterial isolation and purification, LB solid medium was used for culture, and the inoculated medium was incubated at 37 °C; isolation and purification were performed according to the morphological characteristics of bacterial colonies, and pure cultures could be obtained after 1–3 rounds of isolation. The pure cultures were inoculated onto LB slant solid medium and stored at 4 °C for later use, while the pure bacterial cultures were also shake-cultured and amplified in LB liquid medium, with 1 mL of bacterial suspension collected and injected into a cryopreservation tube containing 500 μL of 60% glycerol for long-term preservation at −80 °C. Colony PCR was used for the molecular identification of the purified bacteria: the PCR reaction system consisted of 1 μL of primer 341F (5′-ACTCCTACGGGAGGCAGCAG-3′) and 1 μL of primer 907R (5′-GGACTACHVGGGTWTCTAAT-3′), 2 μL of dNTP, 0.5 μL of Taq enzyme, 2.5 μL of Buffer, and 18 μL of ddH2O, and a colony was added to this reaction system. The PCR amplification was carried out under the following program: initial denaturation at 95 °C for 2 min, followed by 30 cycles of 95 °C for 40 s, 56 °C for 40 s, and 72 °C for 1 min, then a final extension at 72 °C for 5 min, and incubation at 4 °C. After amplification, the PCR products were detected by electrophoresis, which showed a band size of approximately 600 bp. Due to the limited length of this partial 16S rRNA fragment, it can only support reliable identification at the genus level rather than precise species classification. The unpurified PCR products were then sent for sequencing, and the results were aligned against the NCBI database to determine the bacterial genus. Unidentified strains were designated as sp.

### 4.5. Detection of Bacterial Cellulose and Lignin Degradation Capabilities

Four types of culture media were employed to determine the cellulose and lignin degradation capabilities of the bacteria, including one medium for detecting cellulose degradation capability and three media for detecting lignin degradation capability. The bacteria were spot-inoculated at the center of the culture medium using the tip of an inoculating loop, followed by inverted incubation for a fixed number of days before observation, and each medium was tested in triplicate to ensure the reliability of the experimental results.

#### 4.5.1. CMC-Na Agar Medium

CMC-Na agar medium (CMC-Na 15 g, NaCl 5 g, KH_2_PO_4_ 1 g, MgSO_4_ 0.2 g, peptone 10 g, yeast extract 10 g, agar powder 20 g. Distilled water was used to bring the volume to 1 L, the pH was adjusted to 7.0, and autoclaving was performed at 121 °C for 20 min.) was used to evaluate the cellulose degradation capability of the bacteria. After spot-inoculating the bacteria onto the medium, the inoculated medium was incubated at 37 °C for 4 days. Subsequently, the medium was immersed in 1 g/L Congo red dye for 15 min, after which the dye was discarded; the medium was then immersed in 1 mol/L NaCl solution for 20 min, and the solution was discarded afterward. The size of the transparent zone around the bacterial colonies was observed and recorded, which was positively proportional to the cellulase activity of the bacteria.

#### 4.5.2. Sodium Lignosulfonate Agar Medium

Sodium lignosulfonate agar medium (Sodium lignosulfonate 2 g, (NH_4_)_2_SO_4_ 2 g, K_2_HPO_4_ 1 g, KH_2_PO_4_ 1 g, MgSO_4_ 0.2 g, CaCl_2_ 2 g, FeSO_4_ 0.05 g, MnSO_4_ 0.02 g, agar powder 20 g. The above components were dissolved in distilled water and the total volume was adjusted to 1 L. The pH was adjusted to 7.0, and the medium was sterilized at 121 °C for 20 min.) was used for the primary screening of bacterial lignin degradation capability. The bacteria were streak-inoculated onto the medium and incubated at 37 °C for 4 days. The growth of bacterial colonies on the medium indicated that the bacteria could utilize lignin as a carbon source for growth and reproduction.

#### 4.5.3. Aniline Blue Agar Medium

Aniline blue agar medium (Yeast extract 10 g, glucose 20 g, aniline blue 0.1 g, agar powder 20 g. Distilled water was added to make the final volume up to 1 L with natural pH, followed by sterilization at 121 °C for 20 min) was used to detect whether the bacteria screened in the primary step possessed lignin peroxidase (LiP) and manganese peroxidase (MnP) activities. The medium with spot-inoculated bacteria was incubated at 37 °C for 5 days, and the size of the decolorization zone around the colonies was observed. A larger decolorization zone indicated a stronger ability of the bacteria to produce LiP and MnP.

#### 4.5.4. Remazol Brilliant Blue Agar Medium

Remazol Brilliant Blue agar medium (yeast extract 10 g, glucose 20 g, Remazol Brilliant Blue 0.1 g, agar powder 20 g; the medium was diluted to 1 L with distilled water at natural pH and sterilized at 121 °C for 20 min) was used to detect whether the bacteria screened in the primary step had laccase (Lac) activity. The medium with spot-inoculated bacteria was incubated at 37 °C for 5 days, and the size of the decolorization zone around the colonies was observed. The larger the decolorization zone, the stronger the ability of the bacteria to produce Lac.

### 4.6. Detection of the Activity of Cellulose and Lignin Degrading Enzymes in Bacteria

#### 4.6.1. Cellulase Activity Detection

A series of gradient glucose solutions was prepared first. Then, 1 mL of 1% CMC-Na buffer solution was taken and immersed in a 50 °C water bath for 30 min, followed by the addition of 2 mL of DNS reagent and 0.5 mL of gradient glucose solution. The mixture was boiled for 10 min, cooled to room temperature, and the absorbance was measured at 550 nm. A standard curve was plotted with glucose concentration as the horizontal axis and absorbance value as the vertical axis. For the experimental group, 0.5 mL of bacterial supernatant was taken, mixed with 1 mL of 1% CMC-Na buffer solution, incubated in a 50 °C water bath for 30 min, then 2 mL of DNS reagent was added, and the mixture was boiled for 10 min. For the blank control, 1 mL of 1% CMC-Na buffer solution was incubated in a 50 °C water bath for 30 min, followed by the addition of 2 mL of DNS reagent and 0.5 mL of bacterial supernatant, and then boiled for 10 min. After cooling in a cold-water bath, the absorbance of both groups was measured at 550 nm. Finally, the glucose concentration in the supernatant was calculated according to the glucose standard curve. The unit of cellulase activity (U) was defined as the amount of enzyme required to produce 1 mmol of glucose per minute. The enzyme activity per milliliter of enzyme solution (U/mL) was calculated using the following formula: U/mL = (X × V1)/(M × T × V2), where X represents the glucose concentration (mg/mL) calculated from the standard curve, V1 is the volume of the reaction solution (mL), M is the molar mass of glucose, T is the reaction time (min), and V2 is the volume of the supernatant (mL).

#### 4.6.2. LiP Activity Detection

The reaction system was prepared by adding 1 mL of 125 mmol/L sodium tartrate buffer solution, 0.5 mL of 0.16 mmol/L aniline blue solution, and 0.5 mL of bacterial supernatant in sequence; the reaction was initiated by adding 0.5 mL of 2 mmol/L H_2_O_2_ solution. The change in absorbance at 651 nm was measured within the first three minutes. The unit of LiP activity (U) was defined as the amount of enzyme required to increase the OD value by 0.1 per minute. The enzyme activity per milliliter of enzyme solution (U/mL) was calculated using the formula: U/mL = N/(0.1 × T × V2), where N is the change in absorbance at 651 nm within the first three minutes, T is the reaction time (min), and V2 is the volume of the supernatant (mL).

#### 4.6.3. MnP Activity Detection

First, 3.4 mL of 50 mmol/L sodium lactate buffer solution, 0.1 mL of a 1.6 mmol/L MnSO_4_ solution, and 0.4 mL of bacterial supernatant were mixed, and the mixture was preheated at 37 °C for 10 min. The reaction was started by adding 0.1 mL of a 1.6 mmol/L H_2_O_2_ solution, and the change in absorbance at 240 nm was measured within the first three minutes. The unit of MnP activity (U) was defined as the amount of enzyme required to increase the OD value by 0.1 per minute. The enzyme activity per milliliter of enzyme solution (IU/mL) was calculated using the formula: IU/mL = N/(0.1 × T × V2), where N is the change in absorbance at 240 nm within the first three minutes, T is the reaction time (min), and V2 is the volume of the supernatant (mL).

#### 4.6.4. Lac Activity Detection

The reaction system was composed of 3 mL of 200 mmol/L acetic acid buffer, 0.5 mL of 7 mmol/L ABTS solution, and 0.5 mL of bacterial supernatant. The change in absorbance at 420 nm was measured within the first three minutes. The unit of Lac activity (U) was defined as the amount of enzyme required to increase the OD value by 0.01 per minute. The enzyme activity per milliliter of enzyme solution (IU/mL) was calculated using the formula: IU/mL = N/(0.01 × T × V2), where N is the change in absorbance at 420 nm within the first three minutes, T is the reaction time (min), and V2 is the volume of the supernatant (mL).

### 4.7. Activity Detection of Dominant Degrading Enzyme Under Different Culture Conditions

The hull wood powder liquid medium was used as the basic culture medium, and four single variables were set sequentially to explore the optimal culture conditions for the activity of dominant degrading enzymes, with the enzyme activity detected under each variable condition to determine the optimal parameter for each factor.

#### 4.7.1. pH Value

A gradient of pH values (pH5, pH6, pH7, pH8, pH9) was set to detect the activity of the dominant degrading enzyme under different pH conditions, so as to determine the optimal pH value for the maximum activity of the dominant degrading enzyme.

#### 4.7.2. NaCl Concentration

Under the optimal pH condition determined in Section 4.7.1, a gradient of NaCl concentrations (0%, 5%, 10%, 15%, 20%) was set, and the activity of the dominant degrading enzyme was detected under each concentration to obtain the optimal NaCl concentration for enzyme activity.

#### 4.7.3. FeSO_4_ Concentration

On the basis of the optimal pH value and optimal NaCl concentration obtained above, a gradient of FeSO_4_ concentrations (0 mg/L, 500 mg/L, 1000 mg/L, 1500 mg/L, 2000 mg/L) was set, and the activity of the dominant degrading enzyme was detected under each concentration to determine the optimal FeSO_4_ concentration.

#### 4.7.4. PEG400 Concentration

Under the optimal conditions of pH value, NaCl concentration, and FeSO_4_ concentration determined in the previous sections, a gradient of PEG400 concentrations (0%, 2.5%, 5%, 7.5%, 10%) was set, and the activity of the dominant degrading enzyme was detected under each concentration to obtain the optimal PEG400 concentration.

### 4.8. Laboratory Antibacterial Experiment

Each bacterial strain was separately cultured with shaking in liquid medium to obtain a bacterial suspension with an OD600 value of 1. Then, 20 μL of the above bacterial suspension was pipetted and spread evenly on LB solid medium. Four sterile filter paper discs were placed on the medium, and the antibacterial agents and their negative controls were separately dropped onto the filter paper discs. The tested antibacterial agents were 50 mg/mL cinnamaldehyde and 0.5% isothiazolinone K100, which are currently applied for the on-site protection of the Nanhai No. 1 shipwreck. All experiments were conducted in three independent biological replicates to guarantee result stability. The antibacterial effect was finally observed and recorded.

## 5. Conclusions

This study investigated microbial contamination and wood degradation risk of the Nanhai No. 1 shipwreck hull, verified the effectiveness of on-site antibacterial agents, and addressed the research gap regarding the role of bacteria in hull degradation; the results showed that the hull’s microbial community was dominated by bacteria, with Actinomycetota as the dominant phylum (relative abundance 52.78%), and among the seven isolated bacterial strains, *Brevibacterium* sp. (NH.SH-B6) exhibited the strongest wood degradation capacity, with cellulase, LiP, MnP, and lLac activities, and when hull wood was used as the sole carbon source, its dominant degrading enzyme (LiP) achieved maximum activity (0.2299 IU/mL) under conditions of pH = 7, 10% NaCl, 1000 mg/L FeSO_4_, and no PEG400, which is consistent with the hull’s inherent microenvironment, while both 50 mg/mL cinnamaldehyde and 0.5% isothiazolinone K100 showed effective antibacterial activity against the isolated strains, microbial proliferation was attributed to sprinkler blind spots caused by the hull’s complex structure, *Brevibacterium* sp. (NH.SH-B6) dominates microbial degradation of the hull and is significantly influenced by the preservation environment, this study fills relevant research gaps and provides technical guidance for hull protection, and future protection strategies should include long-term microbial community monitoring, optimization of the sprinkler system to reduce blind spots, and targeted regulation of the preservation environment, as this study provides a scientific basis for the protection of the Nanhai No. 1 shipwreck and other similar marine-excavated wooden cultural relics.

## Figures and Tables

**Table 1:** Bacteria isolated from the hull of the Nanhai No. 1 shipwreck.

Strain Number	Genus	Phylum
NH.SH-B1	*Alcaligenes* sp.	Pseudomonadota
NH.SH-B2	*Bacillus* sp.	Bacillota
NH.SH-B3	*Janibacter* sp.	Actinomycetota
NH.SH-B4	*Micrococcus* sp.	Actinomycetota
NH.SH-B5	*Brachybacterium*	Actinomycetota
NH.SH-B6	*Brevibacterium* sp.	Actinomycetota
NH.SH-B7	*Brevundimonas* sp.	Pseudomonadota

**Table 2:** The highest enzyme activity and production time of cellulose and lignin degradation enzymes of NH.SH-B6.

Enzymes	Maximum Enzyme Activity	Generation Time
cellulase	0.0233 IU/mL	36 h
Lip	0.1953 IU/mL	54 h
Mnp	0.0392 IU/mL	54 h
Lac	0.0133 IU/mL	36 h

## Data Availability

The raw sequencing data can be accessed at the NCBI Sequence Read Archive (SRA) under the study accession number PRJNA1015580.
